# Behavioural observation tool for patient involvement and collaboration in emergency care teams (PIC-ET-tool)

**DOI:** 10.1186/s12873-023-00841-7

**Published:** 2023-07-01

**Authors:** Hanna Dubois, Johan Creutzfeldt, Tanja Manser

**Affiliations:** 1 Department of Clinical Science, Intervention and Technology, Karolinska Institutet, K32, Karolinska University Hospital, Stockholm, S-14186 Sweden; 2 FHNW School of Applied Psychology, FHNW University of Applied Sciences and Arts Northwestern Switzerland, Riggenbachstrasse 16, Olten, CH-4600 Switzerland

**Keywords:** Emergency care, Professional-patient relations, Patient participation, Behaviour observation instrument, Instrument development

## Abstract

**Background:**

Patient participation is advocated in various healthcare settings. Instruments for assessment and feedback have been developed to strengthen clinician-patient interaction. In an emergency department context, such instruments are still missing.

The study aimed to develop and test an observation tool for emergency teams’ behaviour regarding patient involvement and collaboration.

**Methods:**

The development of the behavioural observation tool followed a systematic approach. The tool’s content was based on various data sources, i.e., published literature, interview and observational data, and expert consensus. An international expert panel reviewed the content and the rating scale and rated its importance for patient involvement and collaboration in a Delphi process. The feasibility and reliability of the tool were tested by trained observers using video recordings of simulated emergencies. Intraclass correlation (ICC) and Kappa-statistics were performed to test the tool’s inter-rater reliability.

**Results:**

The PIC-ET tool, a 22-item observation instrument was developed in which patient involvement and collaboration behaviours are rated from ‘no’ to ‘high’ using behavioural anchors. Expert agreement was obtained after three Delphi rounds on the tool content, the behavioural anchors and its importance for patient involvement and collaboration.

The content validity was assessed as high, and the tool was found feasible for research. Overall inter-rater reliability was fair (Kappa 0.52).

**Conclusions:**

A novel tool for assessing emergency teams’ behaviour regarding patient involvement and collaboration is introduced. The tool’s psychometric properties were fair to good. Further validation of the PIC-ET tool is recommended for more robust evidence. Future adaptation to different contexts and areas of use, as well as further validity testing may be of value.

**Supplementary Information:**

The online version contains supplementary material available at 10.1186/s12873-023-00841-7.

## Introduction

Emergency care is characterised by short patient-clinician encounters, at times critically ill or severely injured patients and considerable uncertainty for patients as well as the care team. For clinicians, emergency care provides a complex, dynamic work environment. The emergency care staff is faced with patients suffering from a wide range of medical conditions, rapid decisions need to be made by prioritising and communicating within the professional team, all the while respecting patients’ autonomy and supporting patient participation [1, 2]. In various healthcare settings, patient participation is widely advocated [3, 4]. It has been shown empirically to improve patient outcomes, satisfaction, and safety [5–7] and is viewed as an important component of high-quality healthcare [8].

Although clinicians strive to provide the best of care and aim to involve their patients, their actions may not always fully reflect their intentions. Wiman and Wikblad observed an ‘instrumental behaviour’ in staff working with emergency patients, which was explained as a ‘lack of emotional involvement but not insensitivity’ [9]. Knowledge and attitudes among clinicians, as well as time constraints and organisational factors, have previously been reported to influence patient participation [10, 11].

For patients, the situation in emergency care is uncertain as they do not know what to expect or how to act [12]. Elmqvist et al. used the metaphor of a ‘game board with hidden rules’ to illustrate the uncertainty inherent in this situation [12]. In emergency care, patient experiences have been reported to be highly dependent on interactions with the care team concerning communication, information, and involvement [13–15].

Initiatives to improve *patient participation* in emergency care have often focused on shared decision-making [16], which has been claimed as essential to meet ethical obligations towards emergency care patients [17] and has therefore been suggested as the default choice for medical decisions, whenever possible [18]. In addition to shared decision-making, patients receiving emergency care have described other important aspects of patient participation, such as getting attention, being respected, being informed in a manner understandable to the patient, and having a sense of control [14, 15].

However, there is no universal definition of patient participation and related concepts (e.g., patient-centred care, patient engagement) in the literature, and these concepts are often used interchangeably. Common elements in descriptions of the related concepts to patient participation include empathy, respect, engagement, relationship, communication, shared decision-making, holistic focus, individualised focus, and coordinated care [19–21]. In our research, we have been guided by the theoretical framework of Cahill [22], who argued that ‘patient participation’ requires that 1) a relationship between the patient and the clinician exists, 2) the gap of knowledge/information between the patient and the clinician is reduced, 3) the clinician transmits a degree of power to the patient, and 4) the patient takes part in intellectual or physical activities, and 5) benefits from these activities. Cahill also illustrated the hierarchical order of the neighbouring concepts ‘patient involvement’, ‘patient collaboration’, ‘patient participation’ and ‘partnership’ as a pyramid: ‘patient involvement’ and ‘patient collaboration’ form the basic level, ‘patient participation’ constitutes the middle, while ‘partnership’, the ideal and most equal form of a patient-clinician relationship, forms the top.

In this framework ‘patient involvement’ and ‘patient collaboration’ seem similar to ‘patient participation’, but do not meet all defining criteria for ‘patient participation’. Eliciting the patient experience is highlighted as relevant in ‘patient involvement’, but compared to ‘patient collaboration’, ‘patient involvement’ does not stress a two-way information flow or the need of reducing the knowledge gap. ‘Patient collaboration’ implies an engagement in discussions on the patient’s part, but in contrast to ‘patient participation’, ‘patient collaboration’ means co-operation in the care process, while ‘patient participation’ does not necessarily imply an agreement between the patient and the professionals [22].

An important criticism of the concept of patient participation has been that it increases the risk of ‘off-loading’ professional responsibility on a patient [23], which would be considered deeply unethical. Patients in emergency care are sometimes unable to participate actively (e.g., due to pain or a deteriorating condition). However, often this situation may be transient and reversed when the patient feels better. Thus, although many patients may be less capable to participate in an acute phase of illness, they need to be involved by being informed about what is happening and by having an opportunity to express their wishes [12, 14, 15]. We argue that even in a situation in emergency care where full patient participation is not achievable or appropriate, at least as a minimum standard, the foundational level of patient participation, i.e., *patient involvement and collaboration* [22] should be considered.

Enhanced patient participation have been called for decades already, especially in regards to patient safety [8, 24]. But scientific methods to evaluate the level of patient participation are still missing in several domains of healthcare. Previously published instruments for assessing clinician-patient interaction have mainly focused on one-to-one interactions, e.g., consultations in primary care [25–31] or long-term care relationships, such as elderly care [32] and cancer care [33–35]. In emergency settings, however, instruments for assessment of team behaviours that focus on patient involvement and collaboration are still missing, especially with regards to this specific environment that includes medical examinations and/or procedures.

To effectively support clinicians in changing their behaviour towards increased patient involvement and collaboration, interventions such as guidelines or trainings might be developed and tested. Such behaviour change interventions will require a scientifically grounded way to assess the behavioural components of patient involvement and collaboration.

For complex, multidimensional behaviours such as patient involvement and collaboration, reliable and valid performance assessment is challenging, especially in dynamic work settings such as emergency care. One established way for performance assessment is the use of behavioural observation systems that have been developed systematically and can thus serve as a basis for valid and reliable assessment and feedback. Within healthcare behavioural observation systems that can help to identify performance gaps and document behaviour change have successfully been developed in various clinical settings for clinical skills [36, 37] as well as for social and cognitive skills that underpin effective performance [38–41].

The aim of this study was to develop and test a structured observation tool for research purposes of emergency teams’ behaviour regarding patient involvement and collaboration.

## Methods

### Theoretical and methodological frameworks

The general approach for developing behavioural observation systems has been described in a condensed manner by Schmutz et al. [41] and has successfully been applied in different fields of healthcare since. This approach contains the following steps: 1. Developing a draft of the behavioural observation system based on available literature (reviews), guidelines, and other scientific sources by subject matter experts, 2. Establishing expert consensus on the content of the draft observation system by performing a Delphi review, 3. Designing and pilot testing the final behavioural observation system, and 4. Defining item weights, again based on expert consensus.

**Fig. 1:**
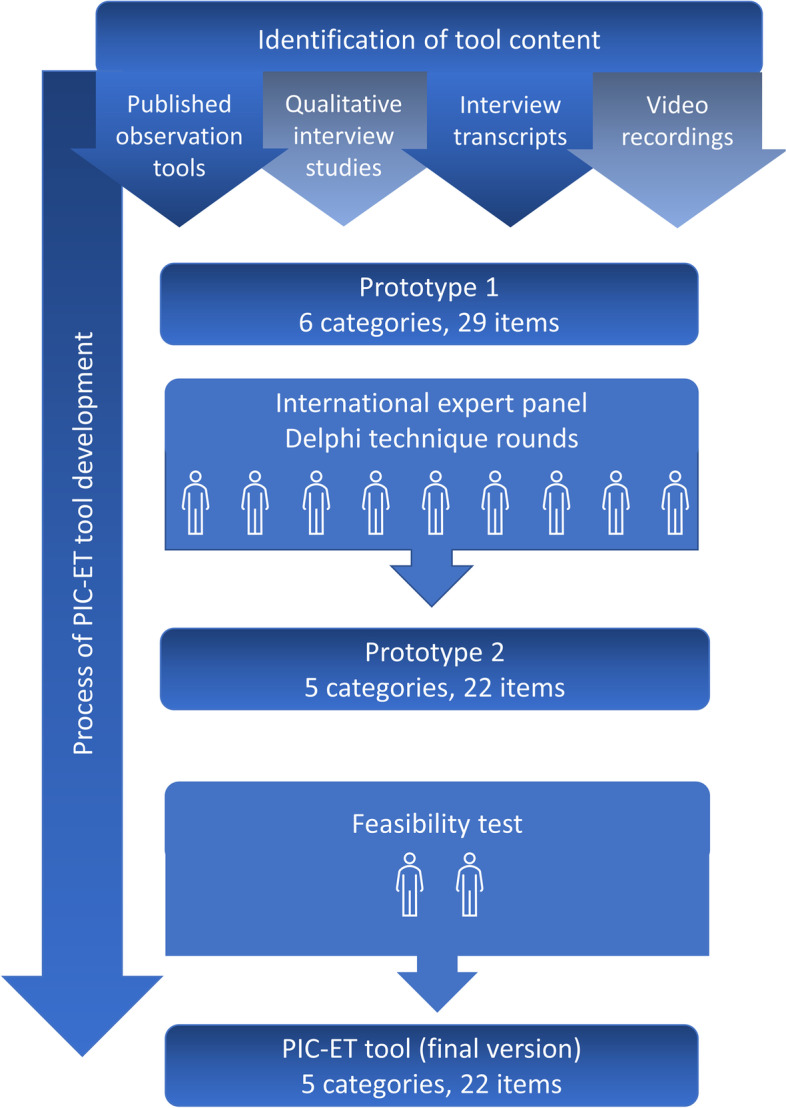
A schematic illustration of the development and testing of the behavioural observation tool for patient involvement and collaboration in emergency care teams (PIC-ET tool)

When developing the content of the observation tool, elements included were inspired by, but not limited to Cahill’s model [22]. Thus, we adopted an inductive approach. Tool development was guided by the systematic methodological approach for developing checklists for clinical performance assessment as described by Schmutz et al. [41] and Brogaard et al. [36]. As recommended by these authors, we drew on various data sources in different steps of the development process such as published literature, interviews and observational data and expert feedback. The development and testing of the tool are illustrated in Fig. 1.

### Development of prototype: content and rating scale

In the first step of the tool content development, we identified observable behaviours by clinicians regarding patient involvement and collaboration in the following data sources:

1. Published observation tools (*n* = 16) for other care settings addressing competencies in the domain of patient participation [25–35, 42–46]. We included the observation tools which were described in a review from 2020 by Ekman et al. [47], and did not find additional observation tools in the literature to include.
2. Qualitative studies representing both the patient perspective [15, 48] and the provider perspective on patient participation [49]. We found it important to incorporate qualitative studies in the content development phase and selected three studies that provided particularly rich descriptions of patient participation from different angles.
3. Transcripts from group interviews (*n* = 13) with health care professionals (*n* = 39) in emergency settings. This interview data was collected within a parallel on-going research project where teamwork and patient participation in emergency teams in northern rural Sweden is being studied. The objective was to evaluate if there are differences between behaviours in teams that are physically co-located and teams that are geographically dispersed and communicating via telemedicine solutions. Descriptions from the interview transcripts of team behaviour related to patient participation in emergency care on a more general level were extracted and used in the content development phase. Behaviours related to the specific ‘tele-emergency’ situation was omitted for this study, however included in a recently published paper [50].
4. Video recordings (*n* = 4) of simulated emergency team training scenarios which were based on real-life emergency cases and reviewed by a multi-professional group of experienced clinicians. This data was also collected within the parallel project described above. The video-recordings are included in other studies that have not yet been published.

The video recordings show multi-professional emergency care teams of three, during in-situ simulation training sessions at their workplace caring for a standardised patient (actor) presenting scripted scenarios of urosepsis or myocardial infarction. In both scenarios, the patient is fully conscious but deteriorating. She expresses opinions and questions about her medical condition, treatment and need for transfer, and displays concern, e.g., about a next of kin who is depending on her.

The second step involved the identification of behaviours, including behaviours both promoting and hindering patient involvement and collaboration. Authors HD and TM inductively searched and charted possible observable behaviours in the different data sources that could be of relevance for the tool development. Then the behaviours were sorted and grouped according to conceptual similarity. The behaviours were consequently thematically grouped into categories and phrased as items capturing the essence of each category. The categories were generally consistent with previous literature on patient participation and its related concepts [19, 20, 22, 51]. For instance, common denominators described in patient participation literature are the trustful relationship, information exchange, and shared decision-making [20, 22, 51] and inspired the naming of items and categories in the observation tool prototype.

To allow for the assessment of team behaviours, behavioural anchors were defined for each item using a scale ranging from ‘no patient involvement and collaboration’ to ‘high patient involvement and collaboration’. The behavioural anchors were inspired by the descriptions that were included in the previously developed chart of observable behaviours.

### Delphi rounds: expert consensus on prototype content and rating scale

A first prototype of the observation tool was submitted to an international expert panel (with members from Sweden, Norway, Denmark, Switzerland, and Australia) in an email-based Delphi technique [52]. The expert panel (Additional file 1) consisted of researchers with expertise in patient participation (*n* = 3), clinical emergency experts (*n* = 3), and patient representatives (*n* = 3). They were identified by approaching a patient association and through our professional networks. The clinical experts and the patient participation experts all had substantial research experience. The three patient representatives all had experience of being patients in emergency departments. Their professional background and medical experiences varied.

**Fig. 2:**
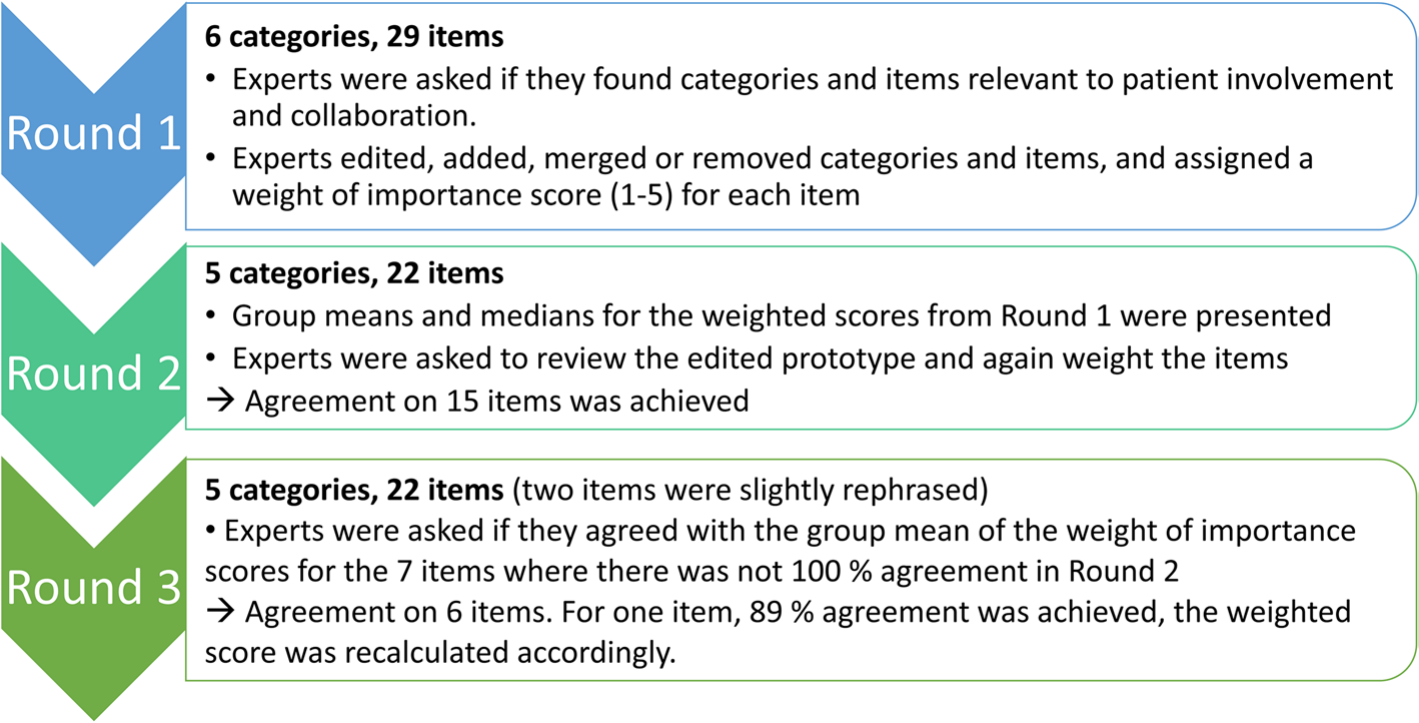
Development of the observation tool prototype by using an expert panel in a Delphi technique procedure

We performed a total of three rounds, where the experts were asked to assess the content and rating scale of the prototype (Fig. 2). The Delphi rounds were mainly e-mail based. However, the patient representatives expressed a need for additional support in completing the Delphi rounds. Thus, their contribution was collected over the phone or by in-person support. The experts were asked to review and revise each category and individual item (content), including the behavioural anchors (rating scale). They were encouraged to comment, remove, add, merge, or edit the content and behavioural anchors of the rating scale in the prototype. During the rounds, all experts remained anonymous to one another, however they all consented to their names being published in the reporting of our work.

### Delphi rounds: expert agreement on the weight of importance scores

In addition to reviewing the content, the items in the prototype were scored by the experts for their weight of importance to patient involvement and collaboration in emergency care on a 5-point Likert scale (1=‘not important at all’, 5=‘very important’). The aim was to achieve agreement on all scores. As a definition of agreement we used Brogaard et al’s model [36] i.e., that agreement is achieved when all scores of the experts’ for one item fall within three neighbouring ratings on the Likert scale.

### Testing prototype feasibility

To test the feasibility of the prototype, we invited two raters (i.e., clinicians with experience in emergency care settings and familiar with the use of behavioural observation tools in research who had not been involved in other parts of the development or testing process) to independently use the prototype to assess two recorded simulated emergency scenarios with a standardised patient (described above). They provided feedback on the phrasing of the items and the rating scales, which informed the last modifications of the prototype. We also asked them how easy it was to use the prototype and to assess the tool’s potential for research.

### Evaluating PIC-ET tool reliability

When the content development, the Delphi rounds and the feasibility testing, were finalised, the prototype was named the PIC-ET tool (patient involvement and collaboration behaviour in emergency teams). To assess the PIC-ET tool’s reliability, two clinicians were selected based on their clinical and research experience of the emergency care setting. They received a half-day rater training where we thoroughly explained and answered questions about each item and the behavioural anchors, and then by practising and discussing the assessment using two video-recorded simulated emergency scenarios (not the same videos used for the actual reliability testing). The reliability raters then used a prototype of the observation tool to rate patient involvement and collaboration in 17 video-recorded simulated emergency scenarios. The videos were 14:09 min in average (range 09:49 min-19:31 min).

### Statistics

Inter-rater agreement was calculated by using statistical software Stata 16.0. Due to the two different types of rating scales, i.e., four or two-level ratings, different indicators for the inter-rater agreement were used.

1. Intraclass Correlation Coefficient (ICC) was calculated for items with four behavioural anchors. Both, absolute agreement and consistency of agreement were calculated by using a two-way random-effects model [53].
2. Cohen’s kappa [54] was calculated for the binary data, i.e., items with two levels, as well as for the categories that included binary items.
3. Per cent agreement was calculated for two of the binary items where Kappa values could not be used, due to lack of variability in the data.

ICC < 0.5 was considered ‘poor’, 0.5–0.75 ‘moderate’, 0.75–0.9 ‘good’, and > 0.9 ‘excellent’ [53]. Kappa values < 0.4 were considered ‘poor’, 0.41–0.6 ‘fair’, and > 0.61 ‘excellent’ [54]. *P*-values < 0.05 were considered significant.

## Results

### First prototype: content and rating scale

A first observation tool prototype was composed based on various data sources and a thematic sorting of observable behaviours related to patient involvement and collaboration (i.e., items, *n* = 29, and categories, *n* = 6). Depending on the content of the item, a rating scale included either four or two behavioural anchors. For most items, a four-level scale was found appropriate.

### Content validity based on expert opinions

In a Delphi technique, where nine experts with different perspectives participated, the first prototype was edited, and the PIC-ET tool took form. The content validity of the tool was assessed. All nine experts completed the three Delphi rounds. The Delphi rounds are illustrated in Fig. 2.

Based on the feedback from the first round, categories and items of the observation tool prototype were edited, merged, or deleted. This revision of the prototype resulted in 5 categories and 22 items. During round 2, additional comments concerning item formulation were made as well, and changes in the prototype were made accordingly. In round 3, after slight revisions, the experts all agreed on the prototype content. Thus, content validity was assessed by the international expert panel: all categories were found relevant for patient involvement and collaboration, and all items were found to fit well in the respective categories. The categories in the final prototype were: ‘Relationship’, Sharing power’, ‘Information Exchange’, ‘Safe and caring environment’, and ‘Social Circumstances’.

### Expert agreement on weight of importance scores

In Delphi round 1, the nine experts assigned all items a ‘weight of importance’ score for patient involvement and collaboration in emergency care (i.e., 1–5), which in round 2 were presented to the experts as group means and medians. In round 2, the weight of importance scores were reviewed and revised, and agreement was reached on 15 of the 22 items’ scores during this round.

**Fig. 3:**
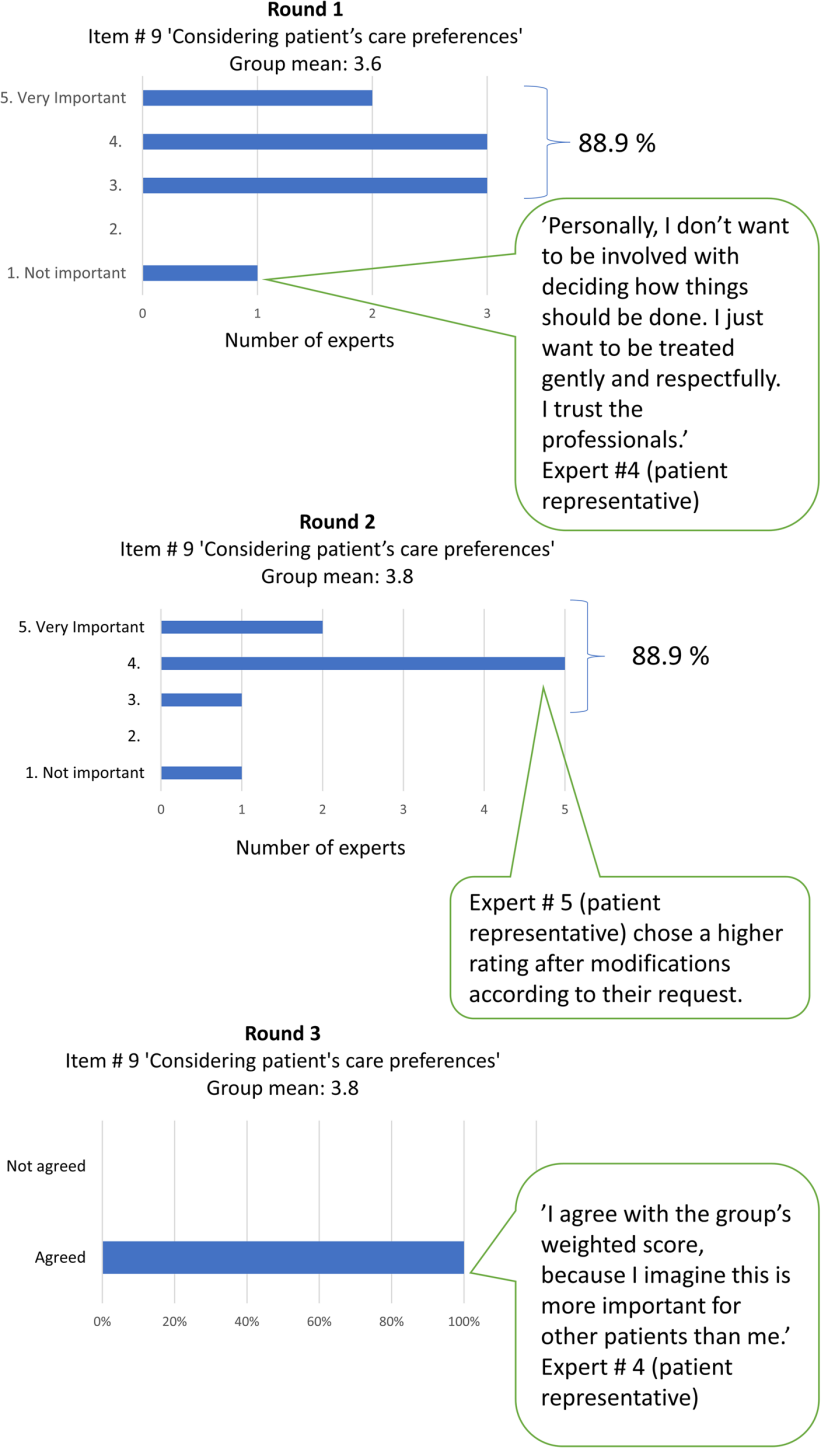
Illustrative examples of the expert agreement on weight of importance scores during Delphi rounds

In round 3, the experts answered whether they could agree with the group means of the weight of importance scores for seven of the remaining items, where agreement had not been achieved in round 2. For one item (# 5 ‘Physical positioning’), agreement was not achieved as one of the experts did not agree on the group mean. Thus, the group mean for that item was recalculated by keeping the disagreeing expert’s rating. Agreement of opinion on the items’ scores between the nine experts was achieved after the three Delphi rounds. The weight of importance scores are presented in Table 1 and the Delphi rounds’ consensus procedure is illustrated in Fig. 3.

**Table 1:** Categories, items, and weight of importance scores in the final PIC-ET tool

Category	Item	Weighted score (1–5, 1 = not important at all, 5 = very important)
Relationship	1. Greeting and team introduction	4.2
	2. Social talk	3.8
	3. Maintaining continuous contact	4.6
	4. Using the patient’s name (avoiding ‘the patient’)	4.0
	5. Physical positioning	3.8
	6.Talking TO and less ABOUT the patient	3.8
	7. Respectful communication	4.9
Sharing power	8. Clarifying with the patient his/her preference concerning the level of information and involvement/collaboration	4.2
	9. Considering the patient’s preferences regarding their medical care	3.8
	10. Involvement in decision-making	4.8
Information exchange	11. Eliciting the patient’s perspective	5.0
	12. Avoiding misunderstanding of information provided by the patient	4.9
	13. Situation updates	4.3
	14. Information and discussion about diagnostics, treatments, procedures, and plan ahead.	4.8
	15. Preparing and supporting through procedures	4.7
Safe and caring environment	16. Safeguarding the patient’s integrity	4.4
	17. Interaction with the patient is well-coordinated within the team	4.2
	18. Optimising physical comfort	4.2
	19. Recognising, acknowledging, and responding to emotions	4.6
Social circumstances	20. Information to next of kin	4.7
	21. Support with practical issues	4.2
	22. Psychosocial issues	3.8

### Feasibility

After having used the tool to assess team behaviour in video-recordings, the feasibility raters provided useful feedback which was implemented by slightly rephrasing two items for clarity, however without changing the meaning or content. As indicated in Table 2, the tool was found quite easy to use and relevant for its different purposes.

**Table 2:** Feasibility raters’ assessments of the utility and relevance of the observation tool prototype

Question (1–5, 1 = not at all, 5 = very well)	Rater 1	Rater 2	Mean
How easy is the instrument to use overall?	4	4	4
How suitable is the instrument for research purposes?	5	5	5
Do you think the necessary ratings can be done based on videos?	4	5	4.5

The feasibility raters had also been encouraged to give us further opinions on the tool, and their reflections from the feasibility testing were used to develop the recommendations for using the PIC-ET tool (Additional file 2).

### Reliability

The results from the reliability evaluation with two independent trained raters indicate ‘fair’ (Kappa 0.52) reliability across all items. Some items reached ‘good’ and ‘excellent’ reliability. Half of the 22 items obtained significant ICC or Kappa values. Reliability for two items (#7 and #8) could not be calculated due to a lack of variability in the data. However, the per cent agreement for these two was 100%.

ICC and Kappa values for categories and items of the observation tool are presented in Table 3.

**Table 3:** Inter-rater agreement: ICC (consistency of agreement and absolute agreement) and Kappa values on categories and items

Categories and items	ICC (consistency of agreement)	CI 95%	*P*-value	ICC (absolute agreement)	CI 95%	*P*- value	kappa	std err	*P*-value
**Relationship (category)**							**0.55**	0.07	< 0.001
1. Greeting and team introduction	**0.97**	0.91 0.99	< 0.001	**0.97**	0.92 0.99	< 0.001			
2. Social talk (Yes/No)							-0.11	0.16	ns
3. Maintaining continuous contact	**0.54**	0.10 0.81	0.01	**0.45**	0.00 0.76	0.01			
4. Using the patient’s name (avoiding ‘the patient’)	**0.89**	0.72 0.96	< 0.001	**0.89**	0.73 0.96	< 0.001			
5. Physical positioning (Yes/No)							-0.06	0.24	ns
6.Talking TO and less ABOUT the patient	**0.66**	0.28 0.86	0.001	**0.61**	0.18 0.84	0.001			
7. Respectful communication (Yes/No)	100% same rating								
**Sharing power (category)**							**0.58**	0.11	< 0.001
8. Clarifying with the patient his/her preference concerning the level of information and involvement/collaboration (Yes/No)	100% same rating								
9. Considering the patient’s preferences regarding their medical care	**0.55**	0.11 0.81	0.01	**0.55**	0.12 0.82	0.01			
10. Involvement in decision-making (Yes/No)							-0.09	0.23	ns
**Information exchange (category)**	**0.45**	0.27 0.61	< 0.001	**0.45**	0.27 0.61	< 0.001			
11. Eliciting the patient’s perspective	0.31	0.00 0.68	ns	0.30	0.00 0.67	ns			
12. Avoiding misunderstanding of information provided by the patient	**0.43**	0.00 0.75	0.04	0.42	0.00 0.74	0.04			
13. Situation updates	**0.51**	0.06 0.79	0.02	**0.50**	0.07 0.78	0.02			
14. Information and discussion about diagnostics, treatments, procedures, and plan ahead.	**0.41**	0.00 0.74	0.04	**0.38**	0.00 0.71	0.04			
15. Preparing and supporting through procedures	0.30	0.00 0.67	ns	0.30	0.00 0.68	ns			
**Safe and caring environment (category)**	**0.47**	0.25 0.65	< 0.001	**0.47**	0.26 0.65	< 0.001			
16. Safeguarding the patient’s integrity	**0.59**	0.08 0.85	0.01	**0.50**	0.00 0.81	0.01			
17. Interaction with the patient well-coordinated within the team	0.29	0.00 0.67	ns	0.28	0.00 0.66	ns			
18. Optimising physical comfort	**0.69**	0.33 0.87	0.001	**0.69**	0.34 0.88	0.001			
19. Recognising, acknowledging, and responding to emotions	0.26	0.00 0.68	ns	0.26	0.00 0.69	ns			
**Social circumstances (category)**							**0.41**	< 0.001	< 0.001
20. Information to next of kin	0.10	0.00 0.54	ns	0.10	0.00 0.54	ns			
21. Support with practical issues (Yes/No)							**0.42**	0.25	0.04
22. Psychosocial issues	0.00	0.00 0.41	ns	0.00	0.00 0.42	ns			
**All items**							**0.52**	0.04	< 0.001

## Discussion

In this paper, we introduce the PIC-ET tool (see Additional file 3) – to the best of our knowledge, the first observation system that focuses on emergency team behaviour related to patient involvement and collaboration. The PIC-ET tool was developed for observing teams caring for an adult patient without severe cognitive impairments. For patients, the emergency setting differs from many other contexts by the sometimes frightening and unpredictable experience and the short-term care encounter with a medical team. As context may matter for the patient’s possibility to be active and involved, the PIC-ET tool has been developed for the contextual factors of emergency settings, including the encounter with a team, instead of a one-to-one clinician-patient consultation.

The PIC-ET tool is divided into five categories and 22 items reflecting key elements of patient involvement and collaboration. It is important to note that the items and categories are often interrelated. For example, item #8 *‘Clarifying with the patient his/her preference concerning the level of information and involvement/collaboration*’ has a natural influence on how item #14 ‘*Information and discussion about diagnostics, treatments, procedures, and plan ahead*’ is handled. Understanding a patient’s preferences and wishes is central to providing opportunities for patient involvement and collaboration [55]. Thus, it should always be done with respect for the patient’s preferences and ability in the moment.

Ekman et al. (2020) reviewed previous assessment tools and checklists within the area of person-centered care [47]. As pointed out by the authors, a majority of the assessment tools lacked a clear conceptual framework to guide the construction of their tools [47]. We relied on the theoretical framework on patient participation, as described by Cahill [22]. By choosing to focus on the foundational levels in this framework, ‘patient involvement and collaboration’, expectations of what can be achieved in an emergency can be kept on a realistic level, without setting an upper limit to patient participation, or even partnership.

Cahill’s theory, as most other theories that include patient involvement and collaboration, was not developed to fit the special context of short-term patient-team encounters. In addition, the emergency setting challenges the conditions for reciprocal interaction. In addition, Cahill’s theory does not describe manifestations of the concept in terms of behaviours. Therefore, we found it necessary to adopt an inductive approach to find relevant content for the tool in this setting.

Another criticism on previously developed checklists and assessment tools within this domain is that patients have not been involved in the development of the instruments [47]. In the PIC-ET tool, the patient perspective was included both by the selection of literature in the early development phase, as well as by including patient representatives in content validation phase.

The development of the PIC-ET tool followed methods described in previous work [36, 41]. The rigorous process of identifying and systematically including team behaviours related to patient involvement and collaboration in different data sources, including perspectives of patients and healthcare professionals, as well as observations of actual emergency teams during simulated patient encounters in an emergency care setting should be viewed as a strength of our study. In addition, an international expert panel contributed to the content development in a Delphi process, which showed promising content validity of the PIC-ET tool. Comments from both the experts and the feasibility raters indicate that the PIC-ET tool has qualities suitable for research purposes.

### Limitations

Our study is not without limitations. Developing an observation tool for interpersonal behaviours in a team context is a complex undertaking. We acknowledge that there are issues that raise questions and may limit the applicability of the PIC-ET tool. At this stage, the PIC-ET tool could be viewed as a promising early version or a pilot.

Although we used international and peer-reviewed literature, as well as an international expert group, the PIC-ET tool was developed and tested in a Swedish context. Therefore attention to assessors’ cultural and contextual competence for the observed setting should be given, as it may impact the perceptions of patient involvement and collaboration. Also, legal requirements, what is considered polite/rude, and what treatment options are available may differ between countries and cultures.

A limitation in our study was the relatively small sample of scenarios and teams included in the development and testing phase. We chose to use simulated scenarios since it is a feasible method and provided us the opportunity to observe and record team behaviour in relation to an emergency patient, without intruding on real patients’ integrity. When designing the cases, we could include opportunities for interaction and patient involvement and collaboration in multiple occasions during the scenarios. We believe that ‘live emergencies’ should be a future step in the validation of this instrument, although this may be more challenging regarding ethical and regulatory aspects to carry out.

We used a Delphi technique in the development and validation of the PIC-ET tool’s content. Delphi groups are often of different sizes and there is no gold standard for the number of experts. Although other studies using expert groups have reported even smaller groups [41, 56], it is fair to say that nine experts is a relatively small group. We however carefully chose the experts and found them highly motivated to provide useful feedback and to complete the rounds. As stated by Boulkedid (2011), a heterogenous expert group can be considered a strength [56]. We found that the experts’ different perspectives enrichened the feedback and contributed to the progress of our work. Also, we assured anonymity between the experts during the Delphi rounds, as this is considered one of the key characteristics of the Delphi technique [57]. Although a larger group could have been valuable, we found the Delphi technique a well-functioning step in the development process.

The reliability test, which was performed on data supplied by two independent raters, showed a ‘fair’ (Kappa 0.52) overall interrater reliability. Although partly inconclusive results, we found the overall interrater reliability as acceptable considering this is a first version of a complex behavioural observation tool. Nevertheless, the reliability assessment of the tool could benefit from further testing, e.g., by using additional clinical scenarios in different settings.

Since this observation tool is newly developed, and this is the first report on its reliability, we suggest caution when using the PIC-ET tool, as its scientific robustness would benefit from being further established. We welcome others to contribute to the validation of this observation tool.

### Future research

For an improved scientific value, the PIC-ET tool’s psychometric properties could be further evaluated. The PIC-ET tool may have the potential for short-term care contexts other than the emergency care setting (e.g., pre-hospital care) and would thus need further testing and potential adaptation in such settings. It could also be argued that patient involvement and collaboration cannot fully be assessed without the contribution of the patient. Thus, another important step in validating the PIC-ET tool would be correlating actual patient experiences to team assessments in ‘live-emergencies’.

Although the aim of the study was to develop an instrument for research purposes, throughout the process it became clear to us that there may be other future potential areas for its use. Patient participation is highlighted in modern curricula in health professionals’ education [58]. Instruments for assessment and feedback for both students and professionals have been developed to raise awareness, ultimately to strengthen patient participation in clinician-patient interaction [25, 42, 43]. Education and training may, thus, be a future area of the PIC-ET tool. The PIC-ET tool could provide an opportunity for an outside view grounded on a 360-degree perspective. The tool could be valuable in education or for feedback on quality improvement projects, as well as a basis for fruitful discussions among clinicians to further improve patient involvement and collaboration. Of course, also for this the PIC-ET tool may need adaptation before validating it in the field of education and training.

We regard the domain of patient involvement and collaboration in emergency settings undertheorized and we present an opportunity to study behaviours in this context, which in turn may contribute to further theoretical development.

## Conclusions

The PIC-ET tool, an instrument for observing emergency care teams’ behaviour for patient involvement and collaboration is introduced for research purposes. The tool has been developed systematically and content validity, as well as feasibility, were found to be high. Overall inter-rater reliability was fair. The scientific value of the PIC-ET tool should be established by further testing and validation. Potential future areas of use for the PIC-ET tool that have been identified are education and training, with the ultimate goal to contribute to improved patient involvement and collaboration in emergency settings.

## Supplementary Information

**Additional file 1.**

**Additional file 2.**

**Additional file 3.**

## Data Availability

The datasets used and analysed during the current study are available from the corresponding author on reasonable request.

## References

[1] Alomari AH, Collison J, Hunt L, Wilson NJ (2021). Stressors for emergency department nurses: insights from a cross-sectional survey. J Clin Nurs.

[2] Davis WD, Dowling Evans D, Fiebig W, Lewis CL (2020). Emergency care: operationalizing the practice through a concept analysis. J Am Assoc Nurse Pract.

[3] Greiner AC, Knebel E (2003). “Institute of Medicine Committee on the Health Professions Education (IOM)”. Health professions education: a bridge to quality.

[4] World Health Organization (2013). Exploring Patient Participation in Reducing Health-Care Related Safety Risks.

[5] McMillan SS, Kendall E, Sav A, King MA, Whitty JA, Kelly F (2013). Patient-centered approaches to health care: a systematic review of randomized controlled trials. Med Care Res Rev.

[6] Olsson LE, Jakobsson Ung E, Swedberg K, Ekman I (2013). Efficacy of person-centred care as an intervention in controlled trials - a systematic review. J Clin Nurs.

[7] Hibbard JH, Greene J (2013). What the evidence shows about patient activation: better health outcomes and care experiences; fewer data on costs. Health Aff.

[8] Institute of Medicine Committee on Quality of Health Care in A (2001). Crossing the Quality Chasm: a New Health System for the 21st Century.

[9] Wiman E, Wikblad K (2004). Caring and uncaring encounters in nursing in an emergency department. J Clin Nurs.

[10] Dyrstad DN, Testad I, Storm M (2015). Older patients’ participation in hospital admissions through the emergency department: an interview study of healthcare professionals. BMC Health Serv Res.

[11] Joseph-Williams N, Elwyn G, Edwards A (2014). Knowledge is not power for patients: a systematic review and thematic synthesis of patient-reported barriers and facilitators to shared decision making. Patient Educ Couns.

[12] Elmqvist C, Fridlund B, Ekebergh M (2012). On a hidden game board: the patient’s first encounter with emergency care at the emergency department. J Clin Nurs.

[13] Blackburn J, Ousey K, Goodwin E (2019). Information and communication in the emergency department. Int Emerg Nurs.

[14] Eriksson-Liebon M, Roos S, Hellström I. Patients' expectations and experiences of being involved in their own care in the emergency department: a qualitative interview study. J Clin Nurs. 2021;30(13–14):1942–52. 10.1111/jocn.15746. 10.1111/jocn.15746 33829575

[15] Frank C, Asp M, Dahlberg K (2009). Patient participation in emergency care - a phenomenographic study based on patients’ lived experience. Int Emerg Nurs.

[16] Flynn D, Knoedler MA, Hess EP, Murad MH, Erwin PJ, Montori VM (2012). Engaging patients in health care decisions in the emergency department through shared decision-making: a systematic review. Acad Emerg Med.

[17] Kraus CK, Marco CA (2016). Shared decision making in the ED: ethical considerations. Am J Emerg Med.

[18] Probst MA, Kanzaria HK, Schoenfeld EM, Menchine MD, Breslin M, Walsh C (2017). Shared Decisionmaking in the Emergency Department: a Guiding Framework for Clinicians. Ann Emerg Med.

[19] Håkansson Eklund J, Holmström IK, Kumlin T, Kaminsky E, Skoglund K, Höglander J (2019). Same same or different?“ a review of reviews of person-centered and patient-centered care. Patient Educ Couns.

[20] Castro EM, Van Regenmortel T, Vanhaecht K, Sermeus W, Van Hecke A (2016). Patient empowerment, patient participation and patient-centeredness in hospital care: a concept analysis based on a literature review. Patient Educ Couns.

[21] Sturgiss EA, Peart A, Richard L, Ball L, Hunik L, Chai TL (2022). Who is at the centre of what? A scoping review of the conceptualisation of ‘centredness’ in healthcare. BMJ Open.

[22] Cahill J (1996). Patient participation: a concept analysis. J Adv Nurs.

[23] Fox A, Reeves S (2015). Interprofessional collaborative patient-centred care: a critical exploration of two related discourses. J Interprof Care.

[24] World Health Organization (2021). Global patient safety action plan 2021–2030: towards eliminating avoidable harm in health care.

[25] Henbest RJ, Stewart MA (1989). Patient-centredness in the consultation. 1: a method for measurement. Fam Pract.

[26] Bertakis KD, Azari R (2011). Determinants and outcomes of patient-centered care. Patient Educ Couns.

[27] Braddock CH, Fihn SD, Levinson W, Jonsen AR, Pearlman RA (1997). How doctors and patients discuss routine clinical decisions: informed decision making in the outpatient setting. J Gen Intern Med.

[28] Chesser A, Reyes J, Woods NK, Williams K, Kraft R (2013). Reliability in patient-centered observations of family physicians. Fam Med.

[29] Elwyn G, Edwards A, Wensing M, Hood K, Atwell C, Grol R (2003). Shared decision making: developing the OPTION scale for measuring patient involvement. BMJ Qual Saf.

[30] Mjaaland TA, Finset A (2009). Frequency of GP communication addressing the patient’s resources and coping strategies in medical interviews: a video-based observational study. BMC Fam Pract.

[31] Shields CG, Franks P, Fiscella K, Meldrum S, Epstein RM (2005). Rochester participatory decision-making scale (RPAD): reliability and validity. The Annals of Family Medicine.

[32] Gaugler JE, Hobday JV, Savik K (2013). The CARES® observational tool: a valid and reliable instrument to assess person-centered dementia care. Geriatr Nurs.

[33] Clayman ML, Makoul G, Harper MM, Koby DG, Williams AR (2012). Development of a shared decision making coding system for analysis of patient–healthcare provider encounters. Patient Educ Couns.

[34] D’Agostino TA, Bylund CL (2014). Nonverbal accommodation in health care communication. Health Commun.

[35] Dong S, Butow PN, Costa DS, Dhillon HM, Shields CG (2014). The influence of patient-centered communication during radiotherapy education sessions on post-consultation patient outcomes. Patient Educ Couns.

[36] Brogaard L, Hvidman L, Hinshaw K, Kierkegaard O, Manser T, Musaeus P (2018). Development of the TeamOBS-PPH - targeting clinical performance in postpartum hemorrhage. Acta Obstet Gynecol Scand.

[37] Schmutz J, Manser T, Keil J, Heimberg E, Hoffmann F (2015). Structured performance assessment in three pediatric emergency scenarios: a validation study. J Pediatr.

[38] Ravindran S, Haycock A, Woolf K, Thomas-Gibson S (2021). Development and impact of an endoscopic non-technical skills (ENTS) behavioural marker system. BMJ Simul Technol Enhanc Learn.

[39] Mitchell L, Flin R, Yule S, Mitchell J, Coutts K, Youngson G (2013). Development of a behavioural marker system for scrub practitioners’ non-technical skills (SPLINTS system). J Eval Clin Pract.

[40] Fletcher G, Flin R, McGeorge P, Glavin R, Maran N, Patey R (2003). Anaesthetists’ non-technical skills (ANTS): evaluation of a behavioural marker system. Br J Anaesth.

[41] Schmutz J, Eppich WJ, Hoffmann F, Heimberg E, Manser T (2014). Five steps to develop checklists for evaluating clinical performance: an integrative approach. Acad Med.

[42] Gallagher TJ, Hartung PJ, Gregory SW (2001). Assessment of a measure of relational communication for doctor–patient interactions. Patient Educ Couns.

[43] Krupat E, Frankel R, Stein T, Irish J (2006). The Four Habits Coding Scheme: validation of an instrument to assess clinicians’ communication behavior. Patient Educ Couns.

[44] Zandbelt LC, Smets EM, Oort FJ, de Haes HC (2005). Coding patient-centred behaviour in the medical encounter. Soc Sci Med.

[45] Paul-Savoie E, Bourgault P, Gosselin E, Potvin S, Lafrenaye S (2015). Assessing patient-centred care for chronic pain: validation of a new research paradigm. Pain Res Manage.

[46] Sabee CM, Koenig CJ, Wingard L, Foster J, Chivers N, Olsher D (2015). The process of interactional sensitivity coding in health care: Conceptually and operationally defining patient-centered communication. J health communication.

[47] Ekman N, Taft C, Moons P, Mäkitalo Ã, Boström E, Fors A (2020). A state-of-the-art review of direct observation tools for assessing competency in person-centred care. Int J Nurs Stud.

[48] Eldh AC, Ekman I, Ehnfors M (2006). Conditions for patient participation and non-participation in health care. Nurs Ethics.

[49] Oxelmark L, Ulin K, Chaboyer W, Bucknall T, Ringdal M (2018). Registered Nurses’ experiences of patient participation in hospital care: supporting and hindering factors patient participation in care. Scand J Caring Sci.

[50] Dubois H, Bergenmar M, Härgestam M, Creutzfeldt J (2022). Patient participation in tele-emergencies - experiences from healthcare professionals in northern rural Sweden. Rural Remote Health.

[51] Nilsson M, From I, Lindwall L (2019). The significance of patient participation in nursing care - a concept analysis. Scand J Caring Sci.

[52] Niederberger M, Spranger J (2020). Delphi technique in Health Sciences: a map. Front Public Health.

[53] Koo TK, Li MY (2016). A Guideline of selecting and reporting Intraclass correlation coefficients for Reliability Research. J Chiropr Med.

[54] Cicchetti DV, Sparrow SA. Developing criteria for establishing interrater reliability of specific items: applications to assessment of adaptive behavior. Am J Ment Defic. 1981;86(2):127–37. 7315877

[55] Nepal S, Keniston A, Indovina KA, Frank MG, Stella SA, Quinzanos-Alonso I (2020). What do patients want? A qualitative analysis of patient, provider, and administrative perceptions and expectations about patients’ hospital stays. J Patient Exp.

[56] Boulkedid R, Abdoul H, Loustau M, Sibony O, Alberti C (2011). Using and reporting the Delphi method for selecting healthcare quality indicators: a systematic review. PLoS ONE.

[57] McKenna HP (1994). The Delphi technique: a worthwhile research approach for nursing?. J Adv Nurs.

[58] Towle A, Farrell C, Gaines ME, Godolphin W, John G, Kline C, et al. The patient's voice in health and social care professional education: the Vancouver statement. Int J Health Governance. 2016;21(1):18–25.

