# Supplementary material for: Behavioural observation tool for patient involvement and collaboration in emergency care teams (PIC-ET-tool)
Source: BMC Emerg Med. 2023 Jul 1;23:74. doi: 10.1186/s12873-023-00841-7 (PMC10314478; doi:10.1186/s12873-023-00841-7)
Supplement: Supplementary file 1 — Additional file 1. [file 12873_2023_841_MOESM1_ESM.pdf]

List of the international expert panel members who contributed during the Delphi rounds in the development of the PIC-ET tool

Experts in patient participation

Mia Bergenmar, nurse, specialist in oncology, Professor, Sophiahemmet University, Stockholm, Sweden

Michelle Kelly, nurse, Professor, University of South Australia, at the time of the project: Associate professor, Curtin School of Nursing, Perth, Australia

Maria Flink, social worker, Associate professor, Karolinska Institutet, Stockholm, Sweden

Clinical emergency experts

Doris Østergaard, physician, specialist in anaesthesiology and intensive care, Professor, University of Copenhagen, Herlev Hospital, Denmark

Monika Brodmann Maeder, physician, specialist in emergency and general internal medicine, Associate professor, Bern University, at the time of the project: University Hospital Inselspital Bern, Switzerland

Torben Wisborg, physician, specialist in anaesthesiology and intensive care, Professor, University of Tromsø, Hammerfest hospital, Finnmark Hospital, Norway

Patient representatives

Angelica Selander, investment advisor, Stockholm, Sweden

Nadja Wedin, artist and pattern designer, Stockholm, Sweden

Pelle Johansson, responsible for research and prevention at The Swedish Heart and Lung Association, Stockholm, Sweden
