# Supplementary material for: Behavioural observation tool for patient involvement and collaboration in emergency care teams (PIC-ET-tool)
Source: BMC Emerg Med. 2023 Jul 1;23:74. doi: 10.1186/s12873-023-00841-7 (PMC10314478; doi:10.1186/s12873-023-00841-7)
Supplement: Supplementary file 2 — Additional file 2. [file 12873_2023_841_MOESM2_ESM.pdf]

Recommendations for use of the PIC-ET tool

Hanna Dubois, Johan Creutzfeldt, Tanja Manser

Stockholm, February 2023

What is the PIC-ET tool?

The PIC-ET tool is an instrument developed to observe teams working in emergency settings in their interaction with an adult patient regarding their behaviour related to patient involvement and collaboration.

The PIC-ET tool was originally designed and tested to be used as a research instrument, to determine levels of patient involvement and collaboration behaviour in emergency teams.

Why do we need it?

Patient involvement and collaboration have been identified as important for emergency patients' experience. Previous research has shown that well informed patients who are involved in decision-making are more satisfied with their care. Also, health outcomes can improve by patient involvement and collaboration. Emergency care is a domain of healthcare where patient involvement and collaboration has been less studied, partly because instruments helping to measure it are missing. Our intention with the PIC-ET tool is to provide a research instrument which can help identify areas of improvement for patient involvement and collaboration, evaluate interventions, and understand interaction between the patient and the professional team in emergencies better – ultimately to improve emergency care and to empower patients who are often in an inferior and vulnerable position.

How should it be used?

This is the first version of the PIC-ET tool, developed by Dubois, Creutzfeldt and Manser (2023). It should be used for observing emergency care teams in their interaction with a patient. The tool is developed for observing teams caring for adult patients without cognitive impairments and who are able to communicate.

The PIC-ET tool contains five categories of behaviour: 1) Relationship, 2) Sharing power, 3) Information Exchange, 4) Safe and caring environment, and 5) Social Circumstances. The assessor can thus identify areas of the team behaviour which may or may not need improvement.

The assessor rates different team behaviours related to patient involvement and collaboration on a rating scale, from *No PIC* (patient involvement and collaboration) to *High PIC*. In the example below, the team behaviour is *Greeting and team introduction*. The levels of patient involvement and collaboration are examples of team behaviours provided to guide the assessor in choosing a level. When behaviours other than the examples are observed, discussions (e.g., between observers and clinicians) are encouraged to choose the corresponding level in the PIC-ET tool.

Levels of patient involvement and collaboration	High	Moderate	Low	No	Area for notes
**1. Greeting and team introduction***<br>*In a large trauma team: team members working close to the patient can be considered as 'all team members', or the team leader can introduce the team.	**All** team members greet and introduce themselves.	**All** team members greet **or** introduce themselves. A few might even do both.	**Some, but not all,** team members greet (e.g., saying 'welcome', 'hello' or waving a hand) **and/or** introduce themselves.	**No one** in the team greets the patient **or** introduces themselves.	

Note that in this tool, the next of kin is not explicitly included, however, communication with a patient may also occur through a next of kin or an interpreter. In these cases, the ratings should be adapted to the situation.

What is the ideal level of patient involvement and collaboration in an emergency?

We do not claim to have an answer to this question. There are many factors that will affect patient involvement and collaboration, such as the medical severity in the situation, the patient's health literacy, language barriers, or even the patient's willingness to participate. The PIC-ET tool may help to identify areas of improvement for the team behaviour. The results from the assessment should always be considered in relation to circumstances of importance. It should, however, be kept in mind that a patient's ability and wish to be involved may change over time.

Considerations for assessors

The assessments when using the PIC-ET tool may be biased if the assessors are influenced by personal beliefs or lack of contextual competence. For research purposes, this is an important consideration, which may require several assessors and possibly group discussions in case of disagreement.

The tool does not have four-level rating options for all items. For some items, it was decided that the frequency of actions would not have improved patient involvement and collaboration, or that rating the quality of actions would bring too much subjectivity to the assessments. In others, e.g., item #7 *Respectful communication*, a four-level rating would have implied that 'a little rudeness' is acceptable. For these reasons, five items in the PIC-ET tool only have two possible ratings. It is still recommended for groups to discuss what is perceived as appropriate behaviour for the situation, even regarding these binary items.

**7. Respectful communication**	**Respectful** communication with or about the patient. Complete absence of comments that are rude, patronizing or judgmental or non-verbal rudeness (e.g., rolling eyes). **Showing an open mind.**		Occurrence of comments to or about the patient that are **rude, patronizing or judgmental**, or non-verbal rudeness (e.g., rolling eyes).

Recommendations for assessor training

When being introduced to the tool, assessors need practice and training by thoroughly talking through each item to avoid misunderstandings, as some of the items may seem very theoretical when reading them for the first time, e.g., how one can clarify with the patient their preferences concerning the level of information and involvement/collaboration (item #8). This practice may preferentially be based on video examples. We suggest at least a half-day of training for assessors familiar with patient participation and emergency settings.

National standards and regulations need to be considered, e.g., when rating involvement in decision-making (item #10), as this in some countries is mandatory in writing and other countries it is not.

Considerations for research

Although the PIC-ET tool was systematically developed, the testing of its reliability for scientific purposes has so far been done in small scale. The psychometric properties of the PIC-ET tool are promising, but the tool may still need development and further validation. When first using it, we recommend caution in interpretations. Ideally, future users may wish to contribute to the development and validation of the PIC-ET tool, perhaps even for other team contexts than the emergency setting.

Contact information

Hanna Dubois
Karolinska Institutet,
Stockholm, Sweden

hanna.dubois@ki.se
