# Supplementary material for: Behavioural observation tool for patient involvement and collaboration in emergency care teams (PIC-ET-tool)
Source: BMC Emerg Med. 2023 Jul 1;23:74. doi: 10.1186/s12873-023-00841-7 (PMC10314478; doi:10.1186/s12873-023-00841-7)
Supplement: Supplementary file 3 — Additional file 3. [file 12873_2023_841_MOESM3_ESM.pdf]

RELATIONSHIP

Levels of patient involvement and collaboration	High	Moderate	Low	No	Area for notes
**1. Greeting and team introduction***<br>*In a large trauma team: team members working close to the patient can be considered as 'all team members', or the team leader can introduce the team.	**All** team members greet and introduce themselves.	**All** team members greet **or** introduce themselves. A few might even do both.	**Some**, **but not all**, team members greet (e.g., saying 'welcome', 'hello' or waving a hand) **and/or** introduce themselves.	**No one** in the team greets the patient **or** introduces themselves.	
**2. Social talk**	**Conversation not related to the visit** (e.g., humor, compliments or small talk) is used to build a trustful relationship between the patient and the team.			All conversation with the patient is **purely medical** or related to the visit.	
**3. Maintaining continuous contact**	There is **always** someone attending to the patient, maintaining contact/conversation and showing interest in what the patient says.	**Most of the time** someone is attending to the patient, maintaining contact/conversation and showing interest in what the patient says. The patient is left unattended only for brief moments.	The patient is left unattended **once or twice** without anybody maintaining contact/conversation or showing interest in what the patient says.	There are frequently **long moments** without anybody maintaining contact/conversation. The team members seem uninterested in what the patient says.	
**4. Using the patient's name (avoiding 'the patient')**	**Always** referring to the patient by his/her name or by talking about 'him/her', never saying 'the patient' as this can be perceived as objectifying.	Referring to the patient by his/her name or by talking about 'him/her' **most of the time**. Only saying 'the patient' once or twice.	Referring to the patient by his/her name **once or twice**, however, saying 'the patient' more often when talking about him/her.	Only referring to the patient as 'the patient'. **Never** using his/her name or talking about him/her.	
**5. Physical positioning**	Somebody in the team makes a **conscious effort** to be at the same physical level with the patient (e.g., by kneeling to be on eye level with the patient or letting the patient sit up) when possible, for medical reasons.			Patient left in a **physically disadvantaged** position (when possibility to higher bed or backrest).	
**6.Talking TO and less ABOUT the patient**	**Consistently**, throughout the visit involving the patient in the team communication by talking TO the patient, not ABOUT the patient; using eye contact, smiles and gestures to invite patient in the discussion.	Involving the patient in the team communication **most of the time** by talking TO the patient.	Involving the patient in the team communication **once or twice** by talking TO the patient.	**No active attempt** to involve the patient in the team communication **or** even **excluding** the patient from the team communication (e.g., 'the nurse is not talking to you; she is talking to the doctor').	
**7. Respectful communication**	**Respectful** communication with or about the patient. Complete absence of comments that are rude, patronizing or judgmental or non-verbal rudeness (e.g., rolling eyes).<br>**Showing an open mind**.			Occurrence of comments to or about the patient that are **rude, patronizing or judgmental**, or non-verbal rudeness (e.g., rolling eyes).	

SHARING POWER

Levels of patient involvement and collaboration	High	Moderate	Low	No	Area for notes
**8. Clarifying with the patient his/her preference concerning the level of information and involvement/ collaboration**	**Yes** (e.g., the patient wants detailed information about findings or does not want to be informed in detail).			**No** (issues about level of information or involvement/collaboration are not at all being addressed)	
**9. Considering the patient's preferences regarding their medical care**	**Several times** asking for or acknowledging patient's thoughts or preferences, **adapting** medical care actions accordingly (e.g., asking in which arm to place iv-catheter) if possible. **Inquiring** about prior experiences.	Asking for/acknowledging patient's thoughts, fears, preferences, allowing for questions **at least once**. **Adapting** medical care accordingly if possible.	**Not asking** patient about his/her thoughts or preferences regarding the medical care, **however being attentive** to and responding to patient's reactions or opinions.	**Never** asking the patient about his/her thoughts or preferences regarding the medical care **or ignoring** preferences stated by patient.	
**10. Involvement in decision-making**	**Involving the patient** in decisions about major care actions/ interventional procedures (e.g., surgery, percutaneous coronary intervention) or non-treatment decisions (e.g., ‘do not resuscitate in case of cardiac arrest’) by discussing possible options/risks and taking into account the patient's wish/consulting next of kin.			**Involving only the medical team** in decisions about major care actions/ interventional procedures (e.g., surgery, per-cutaneous coronary intervention) or non-treatment decisions (e.g., ‘do not resuscitate in case of cardiac arrest’) without considering the patient's wish/consulting next of kin.	

INFORMATION EXCHANGE

Levels of patient involvement and collaboration	High	Moderate	Low	No	Area for notes
**11. Eliciting the patient's perspective**	Inviting/acknowledging the patient to contribute with his/her perspective and understanding of the illness/injury, in his/her own words by using **several open-ended question** (e.g., 'Can you tell me what happened', 'Why do you think that is') and giving **sufficient time** for the patient to respond.	Inviting/acknowledging the patient to contribute with his/her perspective and understanding of the illness/injury, in his/her own words by using **at least one open ended question** and giving **sufficient time** for the patient to respond.	Inviting/acknowledging the patient to contribute with his/her perspective and understanding of the illness/injury, in his/her own words by using **only close-ended questions** (e.g., 'Have you experienced something like this before'), **not giving sufficient time** for the patient to elaborate the answers.	**Not asking** the patient about his/her perspective or understanding on the illness/injury, **or not acknowledging** what the patient tries to tell the team.	
**12. Avoiding misunderstanding of information provided by the patient**	**Consistently** ensuring that complex or crucial information provided by the patient has been correctly understood (e.g., by repeating or by asking adequate follow-up questions).	**Frequently** ensuring that complex or crucial information provided by the patient has been correctly understood.	**Once or twice** ensuring that complex or crucial information provided by the patient has been correctly understood.	**Never** ensuring that complex or crucial information provided by the patient has been correctly understood.	
**13. Situation updates**	**Regularly** giving situation updates directly to the patient or to the entire team, without using medical jargon so that the patient can follow (e.g., next step, clinical workflow, reason for waiting).	Giving situation updates understandable to the patient **once or twice** (e.g., next step, clinical workflow, reason for waiting).	Only giving situation updates (e.g., next step, clinical workflow, reason for waiting) **upon patient's request**, or by using **too much medical jargon** for the patient to follow.	**Never** giving any situation updates (e.g., next step, clinical workflow, reason for waiting).	
**14. Information and discussion about diagnostics, treatments, procedures, and plan ahead.**	Information (e.g., lab results, findings) and suggestions for procedures, treatments, and plan ahead are presented to the patient in an **understandable manner** (not using medical jargon). **Checking** patient understanding. **Inviting** the patient to discuss. The patient is **given time** to react to and absorb the information/ discussions.	Information (e.g., lab results, findings) and suggestions for procedures, treatments and plan ahead are presented to the patient in an **understandable manner** (not using medical jargon). Discussions with the patient **only** occur on the **patient's initiative**.	Being **transparent** about findings and plans (e.g., thinking out loud, mentioning lab results) but **not adapting** language so that a layperson can understand. **Informing rather than discussing** with the patient.	All information and discussions about medical findings and plans are **only** shared **between the professionals**.	
**15. Preparing and supporting through procedures**	**Thoroughly** preparing (e.g., what to expect) and supporting (e.g., breathing techniques, holding hands) the patient through procedures (e.g., ultrasound, lumbar puncture). **Allowing** for questions, **telling** the patient that he/she can ask for the procedure to be paused or discontinued.	**Briefly** preparing (e.g., 'this may hurt') and supporting (e.g., breathing techniques, holding hands) the patient through procedures (e.g., ultrasound, lumbar puncture).	Performing procedures **only** by briefly preparing and supporting the patient **after prompts** from the patient.	Performing procedures **without** preparing or supporting the patient.	

SAFE AND CARING ENVIRONMENT

Levels of patient involvement and collaboration	High	Moderate	Low	No	Area for notes
**16. Safeguarding the patient's integrity**	**At all times**, patient's integrity is safeguarded. Time exposing the patient's body is **minimized** (e.g., putting a blanket over her/him when undressed for ECG) and is **always** proceeded by asking for permission.	**Most of the time**, only missing to safeguard patient's integrity on a few occasions. However, some **room for improvement** (e.g., minimizing spectators, drawing curtains).	**Some of the time**, but clearly missing to safeguard patient's integrity at **important moments** (e.g., undressing without asking or leaving patient exposed longer than necessary).	**Not at all**.	
**17. Interaction with the patient is well-coordinated within the team**	All parts of interaction with the patient are **highly coordinated** within the team (e.g., transitions between tasks involving the patient are smooth, the patient is being addressed by one person at a time). **Consistency** in information given to the patient from different team members. Calm, yet professional ambiance within the team.	**Most of the time**. Some moments of uncoordinated interaction with the patient (e.g., simultaneous talking within the team). **Consistency** in information given to the patient from different team members.	**Some of the time**. Some efforts of coordinating interaction with the patient within the team. **Some inconsistency** in information given to the patient from different team members.	Interaction with the patient **uncoordinated** within the team (e.g., excessive and simultaneous talking). **Inconsistency** in information given to the patient from different team members.	
**18. Optimising physical comfort**	**Frequently** checking on patient and asking about physical comfort, responding to patient's needs by offering help (e.g., patient positioning, providing blanket or pillow).	Asking about patient's physical comfort and responding to patient's needs by offering help **at least once** (e.g., 'Are you cold, do you need a blanket?').	Optimizing physical comfort in response to **patient request** (e.g., patient asking for a blanket).	**Not at all**. Not responding even if patient expresses discomfort.	
**19. Recognising, acknowledging, and responding to emotions**	**At all times**, verbally (e.g., calming words if the patient is anxious) **or** non-verbally (e.g., expressive touch to let the patient know that you care) recognising and responding to patient's emotions (e.g., 'It is normal to feel afraid' or 'I can see you are sad'). **Inquiring** about feelings if patient is not expressing emotions (e.g., 'How does this it make you feel?').	**Most of the time**, all clear expression of emotion by the patient (e.g., crying) is responded to.	**Some of the time**, but clearly not responding to several clear expressions of emotion by the patient.	**Not at all**.	

SOCIAL CIRCUMSTANCES

Levels of patient involvement and collaboration	High	Moderate	Low	No	Area for notes
**20. Information to next of kin**	**Offering** to inform the next of kin according to the **patient's wishes**. **Inviting** the patient's input regarding who to inform, in what way or, if possible, giving the patient the **opportunity** to talk to next of kin directly.	**Offering** to inform next of kin.	Informing next of kin upon **patient request**.	**Not prioritising** information to next of kin even when the patient asks for it.	
**21. Support with practical issues**	**Supporting** the patient in dealing with practical issues (e.g., charging phone, handing over home keys to neighbor, calling friends or family to check on pets).			**Not offering** help with practical issues.	
**22. Psychosocial issues**	**Asking** about, **recognizing** and **adapting** care plan as much as possible to the patient's psychosocial issues (e.g., living situation, work, domestic violence).	Taking psychosocial issues into consideration **only after prompts** from patient.	**Listening** to the patient when he/she brings up topics related to psychosocial issues. However **not adapting** care plan accordingly and not explaining why.	**Ignoring** or **devaluating** patient's psychosocial issues.	
